# Persistent Hypoglossal Artery as a Potential Risk Factor for Simultaneous Carotid and Vertebrobasilar Infarcts

**DOI:** 10.3389/fneur.2018.00837

**Published:** 2018-10-11

**Authors:** Xingyi Jin, Libo Sun, Zheng Feng, Xiaodong Li, Hongyan Zhang, Ke Meng, Weidong Yu, Chao Fu

**Affiliations:** ^1^Department of Neurosurgery, China-Japan Union Hospital of Jilin University, Changchun, China; ^2^Department of Pediatrics, China-Japan Union Hospital of Jilin University, Changchun, China; ^3^Department of Neurosurgery, Siping Central People's Hospital, Siping, China; ^4^Department of Neurology, China-Japan Union Hospital of Jilin University, Changchun, China

**Keywords:** persistent hypoglossal artery, carotid artery, vertebrobasilar artery, atherosclerosis, ischemia, risk factor

## Abstract

Persistent hypoglossal artery (PHA), a rare embryological carotid–basilar anastomosis, is usually accompanied by hypoplastic vertebral and posterior communicating arteries, and thereby such vascular anomaly serves as the main feeder supplying the vertebrobasilar territory. Although rarely reported, simultaneous anterior and posterior territory infarcts related to PHA and carotid atherosclerosis can occur. To date, as far as we know, only 4 such cases have been previously reported in the literature. Here, we present the case of a 65-year-old female with a PHA and carotid atherosclerotic plaques, who developed acute multiterritorial infarcts involving the left carotid and vertebrobasilar territories. This case highlights that such a persistent anastomosis should be considered when multiple infarcts involving the anterior and posterior territories are encountered, and should be kept in mind when dealing with carotid atherosclerotic lesion.

## Introduction

Hypoglossal, trigeminal, optic, and proatlantal arteries form anastomoses between the carotid artery and the basilar artery in the fetal circulation (1–7). Carotid-vertebrobasilar anastomoses usually disappear during embryogenesis. Persistent hypoglossal artery (PHA) is a rare carotid–basilar anastomosis, with a reported angiographic prevalence of 0.03–0.09% (1). It usually arises from the internal carotid artery (ICA) between the C1 and C3 levels, whereas rarely originates from the external carotid artery. It traverses through the hypoglossal canal to join the vertebrobasilar system. Generally, the existence of PHA is associated with hypoplastic bilateral vertebral arteries, and in such cases, PHA serves as the main source of blood supply to the vertebrobasilar territory.

PHA is found incidentally in most of the reported cases; however, it can be as the potential pathway of emboli from the heart or proximal ICA to reach the posterior circulation, leading to ischemic stroke (1–3). Notably, embolic infarcts, although rare, can occur simultaneously in both the anterior and posterior circulation territories in patients with PHA and carotid atherosclerotic disease. To the best of our knowledge, only 4 such cases have been reported (3–5).

We herein presented a case of a PHA in a 65-year-old woman with carotid atherosclerosis, who developed acute multiterritorial embolic infarcts involving the left carotid and vertebrobasilar territories.

## Case presentation

**Figure 1:**
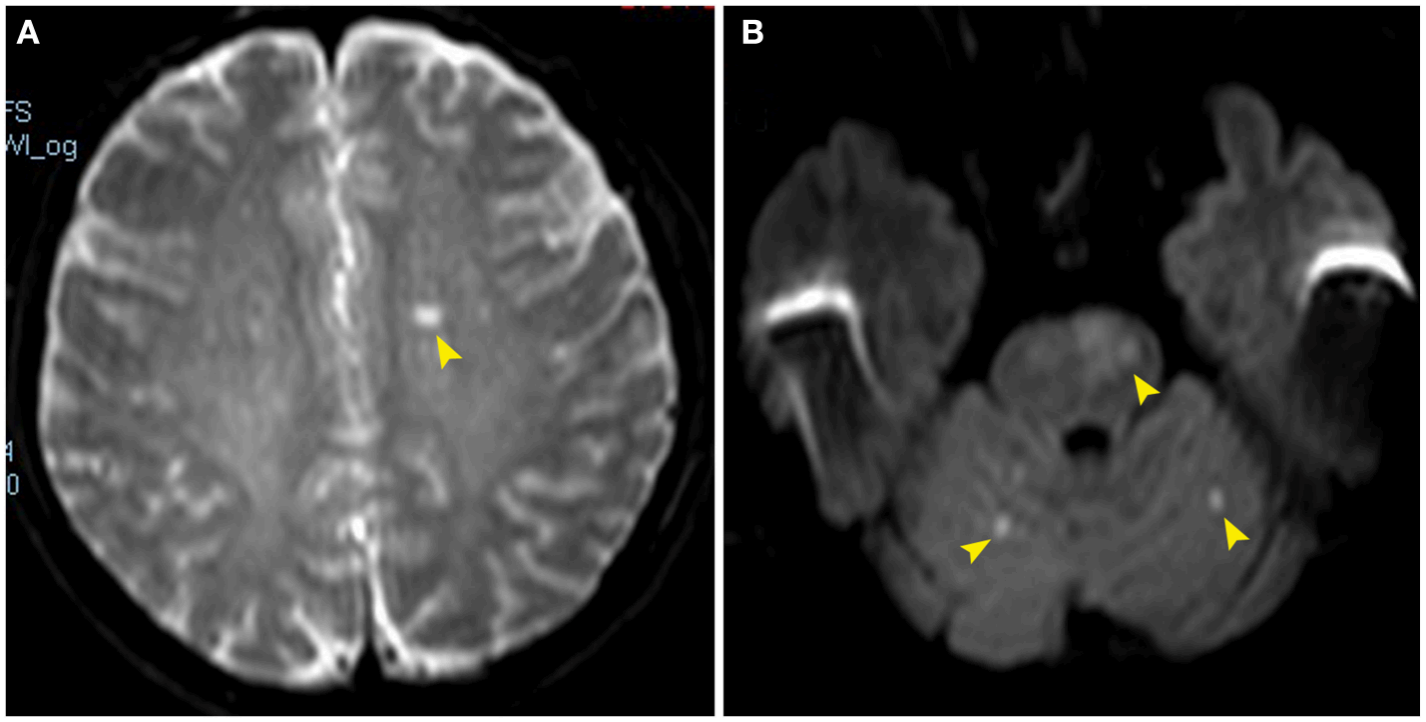
Diffusion-weighted magnetic resonance images showing acute infarcts involving the left corona radiate (**A**, arrowhead), bilateral cerebellar hemispheres and left pons (**B**, arrowheads).

**Figure 2:**
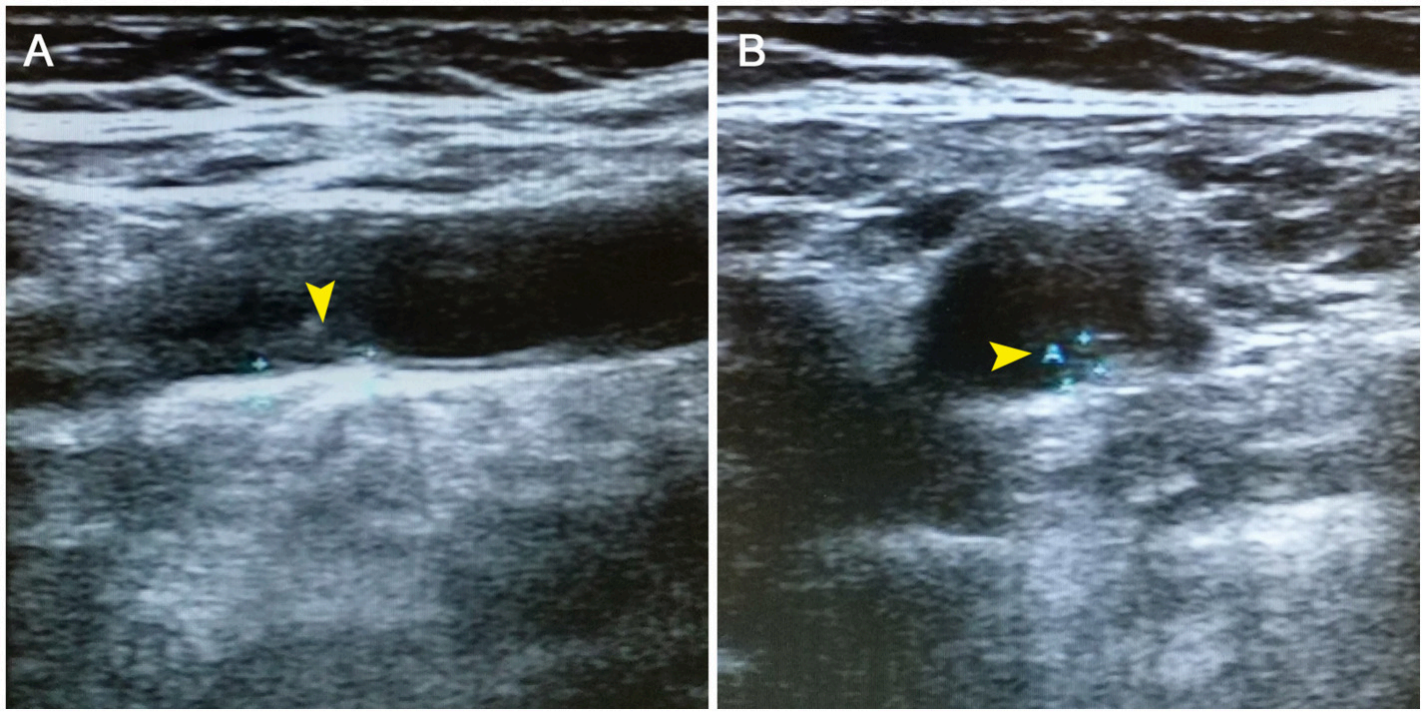
Cervical color doppler ultrasonography revealing atherosclerotic plaques (**A**,**B**, arrowheads) of the left internal carotid artery.

**Figure 3:**
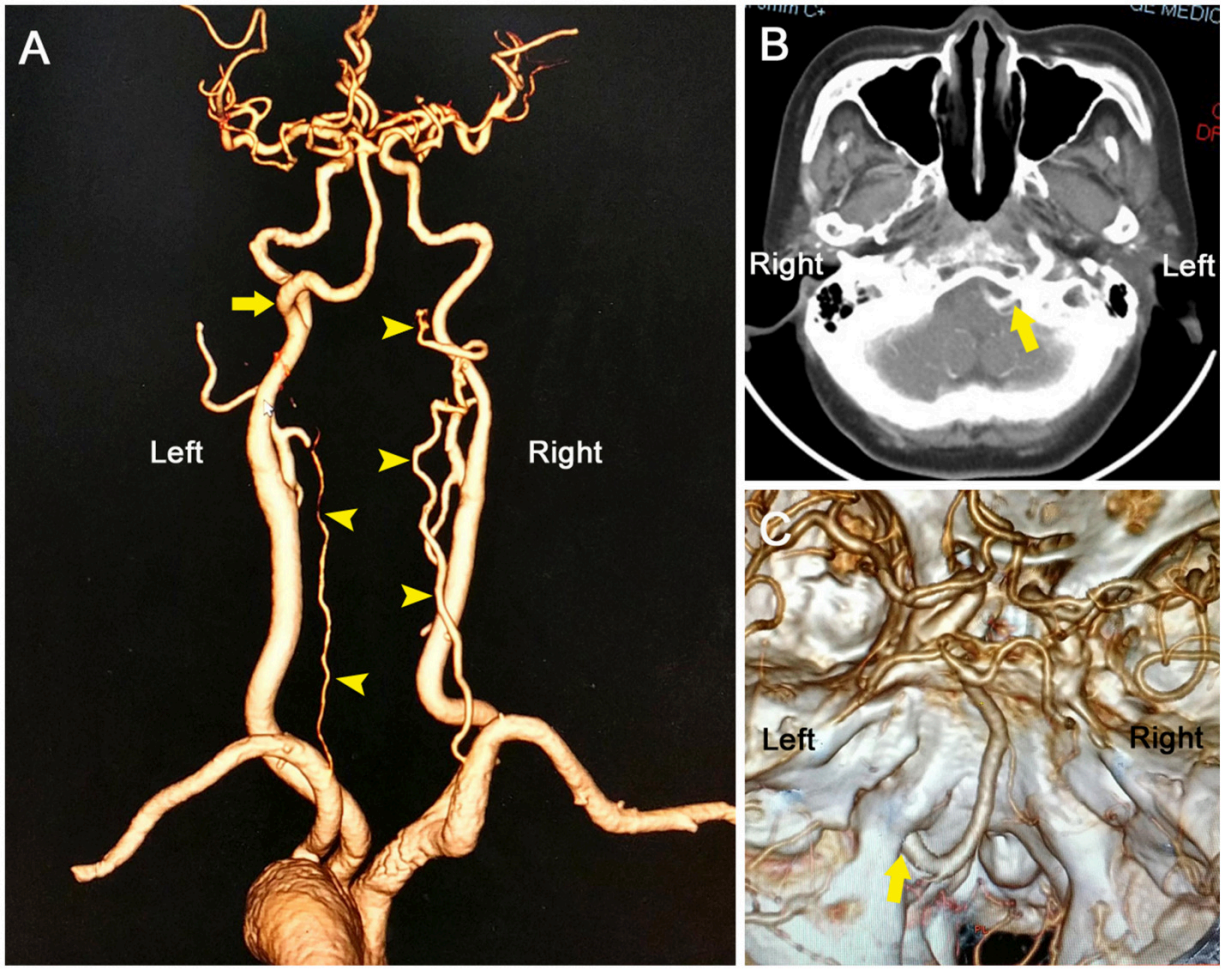
Computed tomography angiography showing an anomalous vessel **(A**, arrow) arising from the left internal carotid artery and then traveling through the left hypoglossal canal **(B,C)**, consistent with the diagnosis of persistent hypoglossal artery. Note that bilateral vertebral arteries were hypoplastic (arrowheads), and no posterior communicating arteries were found; therefore, the PHA primarily fed the posterior circulation.

A 65-year-old female presented with dizziness of 3 days duration. She had no history of hypertension, diabetes mellitus, and cardiovascular disease. Admission neurological examination was unremarkable. Electrocardiography showed no atrial fibrillation and transesophageal echocardiography showed no anomaly. Non-contrast head computed tomography (CT) demonstrated no significant infarction. Diffusion-weighted magnetic resonance imaging revealed acute multiterritorial infarcts in the left corona radiata, bilateral cerebellar hemispheres, and left pons (Figure 1). Cervical color ultrasound examination demonstrated vulnerable atherosclerotic plaques at the origin of the left ICA (Figure 2), and bilateral low flow with a high resistance flow pattern in both vertebral arteries while normal flow in the basilar artery. CT angiography of the head showed a left PHA, bilateral hypoplastic vertebral arteries, and no posterior communicating arteries (Figure 3). The PHA arose from the cervical segment of the left ICA, ran upward, took a somewhat tortuous course, and continued as the ipsilateral vertebral artery through the left hypoglossal canal, thereby serving as the major contributor of the posterior circulation. Aspirin (100 mg/day) and atorvastatin (20 mg/day) combination therapy was instituted, and her neurological condition remained stable during 6-months follow-up.

## Discussion

PHA is the second most common persistent embryological carotid-vertebrobasilar anastomosis (0.03–0.09%), after persistent trigeminal artery (0.1–0.6%) (1, 6). Numerous associated anomalies have been reported with PHA such as cerebral ischemia, cerebral aneurysm, arteriovenous malformation, and moyamoya disease (1–8). Their significance is unclear. Generally, PHA is a clinically silent anatomical variation. Glossopharyngeal neuralgia and hypoglossal nerve palsies have been reported as rare manifestations of a persistent hypoglossal artery.

PHA may be of clinical importance in particular circumstances such as the one described in our case. In our patient, PHA served as the major supply to posterior circulation because vertebral and posterior communicating arteries were hypoplastic or aplasic. Although PHA is usually an incidental finding in the majority of cases, this vascular anomaly can act as a potential risk factor of embolism in patients with carotid atherosclerosis or atrial fibrillation.

Simultaneous anterior and posterior circulation infarctions associated with carotid atherosclerosis and PHA is rare. To date, only 5 such cases, including the present case, have been reported in the English-language literature (Table 1) (3–5, 9). It is worth noting that most patients had bilateral hypoplastic vertebral arteries. To our knowledge, this is the first report of absent posterior communicating arteries related to PHA in patient with carotid atherosclerosis disease. Two patients were treated with carotid endarterectomy, and 3 received medical management. These patients' symptoms could be attributed to embolic phenomenon from atherosclerotic plaque of the ipsilateral carotid artery, blood supply insufficiency due to carotid stenosis or both.

**Table 1:** Reported cases with simultaneous carotid and vertebrobasilar infarctions related to PHA and carotid atherosclerotic lesions.

**Year**	**Authors**	**Sex/age (years)**	**Symptom**	**Infarcts**	**Atherosclerotic lesion**	**PHA**	**VAs**	**PComA**	**Treatment**	**Outcome**
1978	Sunada et al. (4)	M/62	Left hemiparesis (4/5), left face hypesthesia	Right corona radiata and occipital lobe	Stenosis extending from the right ICA to the PHA	Right	Hypoplastic	?	Endarterectomy	?
2007	Pyun et al. (3)	F/76	Recurrent Vertigo, transient left-sided motor weakness	Right frontal lobe and bilateral cerebellar hemispheres	Stenosis of the right proximal ICA	Right	Hypoplastic	Hypoplastic	Aspirin + clopidogrel	?
2009	Kawabori et al. (5)	M/73	Consciousness disturbance	Right frontal cortex, bilateral occipital lobes, bilateral cerebellar hemispheres, and brainstem	Stenosis of the right proximal ICA	Right	Hypoplastic	?	Endarterectomy	Good
2018	Han et al. (9)	F/82	Dizziness, left hemiparesis	right cerebral hemisphere, bilateral occipital lobe, bilateral cerebellar hemispheres	carotid artery dissection of the right proximal ICA	Right	?	?	Anticoagulation + antiplatelet	?
2018	Present study	F/65	Dizziness	Left corona radiata, bilateral cerebellar hemispheres and pons	Vulnerable atherosclerotic plaques of the left proximal ICA	Left	Hypoplastic	Absent	Aspirin + atorvastatin	Good

*M, male; F, female; ICA, internal carotid artery; PComA, posterior communicating artery; PHA, persistent hypoglossal artery; VA, vertebral artery; ?, not stated*.

In this case, the etiology of infarcts were considered to be artery-to-artery embolism on the basis of the following remarks: (a) vulnerable atherosclerotic plaques at the proximal left ICA were visible as the presumed source of emboli; (b) simultaneous multi-territorial infarctions in the carotid and vertebrobasilar territories were most likely to be correlated with embolism; (c) the PHA originated from the ipsilateral ICA and was the predominant vascular supply to the posterior circulation; (d) cardiac embolism was less likely although it could not be excluded as no holter monitoring nor extended cardiac monitoring was performed.

The presence of PHA represents a significant therapeutic challenge when dealing with carotid atherosclerotic disease. Clamping of the ICA during carotid endarterectomy may result in such case in a significant decrease of cerebral perfusion in the anterior as well as the posterior circulation (10). Carotid artery stenting presented a risk for distal artery embolism, and thus it is necessary to deploy two distal protection devices within the distal ICA and distal PHA to protect the ipsilateral anterior and posterior vessels from emboli (11).

## Conclusions

Although rare, PHA should be considered when simultaneous anterior and posterior territory infarcts were encountered. Such a persistent anastomosis is a potential risk factor for ischemic stroke, and should be kept in mind in particular during carotid endarteriectomy or stenting for carotid atherosclerotic diseases.

## Ethics statement

This study was carried out in accordance with the recommendations of the Ethics Committee of the China-Japan Union Hospital of Jilin University with written informed consent from all subjects. Written informed consent was obtained from the participant for the publication of this case report.

## Author contributions

XJ, LS, and ZF were involved in the case selection, in addition to planning the manuscript. XL, HZ, KM, and WY were involved in the case discussion. CF contributed to the planning, manuscript drafting and revision, and final approval.

### Conflict of interest statement

The authors declare that the research was conducted in the absence of any commercial or financial relationships that could be construed as a potential conflict of interest.
